# Early sexual activity lowers the incidence of intracranial aneurysm: a Mendelian randomization investigation

**DOI:** 10.3389/fneur.2024.1349137

**Published:** 2024-06-04

**Authors:** Pengfei Wu, Paziliya Akram, Kaheerman Kadeer, Maimaitili Aisha, Xiaojiang Cheng, Zengliang Wang, Aierpati Maimaiti

**Affiliations:** Department of Neurosurgery, Xinjiang Medical University Affiliated First Hospital, Ürümqi, Xinjiang, China

**Keywords:** sexual behavior, intracranial aneurysm, Mendelian randomization, subarchinoid hemorrhage, sexual intercourse

## Abstract

**Objective:**

Investigate the potential correlation between the age of initial sexual contact, the lifetime accumulation of sexual partners, and the occurrence of intracranial aneurysm (IA) employing a two-sample Mendelian randomization approach.

**Methods:**

This research aims to elucidate the causal relationship between intracranial aneurysm (IA) and sexual variables. Two distinct sexual variables, specifically the age had first sexual intercourse (*n* = 406,457) and the lifetime number of sexual partners (*n* = 378,882), were employed as representative parameters in a two-sample Mendelian randomization (MR) study. Outcome data from 23 cohorts, comprising 5,140 cases and 71,934 controls, were gathered through genome-wide association studies (GWAS). To bolster analytical rigor, five distinct methodologies were applied, encompassing MR-Egger technique, weighted median, inverse variance weighted, simple modeling, and weighted modeling.

**Results:**

Our investigation unveiled a causal relationship between the age first had sexual intercourse and the occurrence of intracranial aneurysm (IA), employing the Inverse Variance Weighted (IVW) approach [Odds Ratio (OR): 0.609, *p*-value: 5.684E-04, 95% Confidence Interval (CI): 0.459–0.807]. This association was notably significant in the context of unruptured intracranial aneurysms (uIA) using the IVW approach (OR: 0.392, *p*-value: 6.414E-05, 95% CI: 0.248–0.621). Conversely, our findings did not reveal any discernible link between the lifetime number of sexual partners and the occurrence of IA (IA group: OR: 1.346, *p*-value: 0.415, 95% CI: 0.659–2.749; SAH group: OR: 1.042, *p*-value: 0.943, 95% CI: 0.338–3.209; uIA group: OR: 1.990, *p*-value: 0.273, 95% CI: 0.581–6.814).

**Conclusion:**

The two-sample Mendelian Randomization (MR) study presented herein provides evidence supporting a correlation between the age of initial engagement in sexual activity and the occurrence of intracranial aneurysm (IA), with a noteworthy emphasis on unruptured intracranial aneurysms (uIA). Nevertheless, our investigation failed to establish a definitive association between IA and the cumulative lifetime number of sexual partners.

## 1 Introduction

Intracranial aneurysm (IA) denotes a medical condition marked by atypical, localized dilations of cerebral arteries, carrying a risk of rupture. This category encompasses both ruptured aneurysms and unruptured intracranial aneurysms (uIA). Rupture poses a substantial risk of spontaneous subarachnoid hemorrhage (SAH), accounting for approximately 85% of such cases (1). A comprehensive global study reported a prevalence of around 3.2% for IA, with participants having an average age of 50 years. Aneurysmal subarachnoid hemorrhage (aSAH) is frequently linked to an unfavorable prognosis, with heightened rates of functional impairment and mortality (2). Despite these clinical implications, the underlying etiopathology of intracranial aneurysms remains elusive.

The prognosis following aneurysmal subarachnoid hemorrhage has demonstrated a modest improvement in recent generations. The incidence of SAH has experienced a marginal decline, and concurrently, the mean age of affected individuals has increased by approximately 10 years. Monique Vlak and colleagues documented the prevalence, determinants, and rupture of aneurysms in 2011 in The Lancet Neurology. The findings did not reveal significant changes in the occurrence of unruptured intracranial aneurysms (uIA) over time. The combined estimated frequency was 3.2% in a demographic with an average age of 50 years, featuring an equal distribution of 50% males and individuals free from comorbidities. However, prevalence was elevated in specific subgroups, such as individuals with autosomal dominant polycystic kidney disease (ADPKD) or a familial background indicating a history of SAH or IA. In a recent study, 24 uIA were detected in 23 out of 461 screened first-degree relatives (FDRs). Following a median follow-up span of 2 years, no changes were observed in any of the unruptured intracranial aneurysms (uIA). After a median follow-up span of 24 months, stability was noted in all instances of uIA. The anticipated likelihood of detecting unruptured intracranial aneurysms (uIA) ranged from 2.3 to 14.7%, with a higher prevalence observed among first-degree relatives (FDRs) who engaged in smoking and substantial substance consumption (3). Across all survey points, health-related quality of life (QoL) and emotional well-being demonstrated similarities to a reference group drawn from the broader population. Although a history of smoking was not included as a determinant of aneurysm instability in the PHASES score, predictive artificial intelligence models suggested an association between smoking and aneurysm instability (4). Adolescence is often perceived as a phase marked by increased participation in behaviors with potential risks, such as precarious sexual activities, deliberate self-harm, and engagement with substances. Studies have indicated a correlation between heightened alcohol intake, early initiation of sexual encounters, and an increased number of sexual partners during this developmental period (5, 6). Despite research suggesting a higher likelihood of developing intracranial aneurysms among individuals who smoke, the connection between intracranial aneurysms and risky sexual factors remains undisclosed.

Comprehensive epidemiological cohort investigations, which demand substantial time, prompt inquiries into the potential impact of risky sexual activities on IA. Consequently, there is a demand for alternative methods to augment causal inference before committing to potentially costly trials. Mendelian Randomization (MR) research provides a methodology for discerning whether exposure influences the outcome. This is achieved by employing large-scale integrated genetic markers as instrumental variables (IVs) to explore the relationships between a given factor and the observed outcomes (7). The robustness of MR research lies in genetic variants demonstrating a certain degree of independence from behaviors selected by individuals and being established well in advance of disease onset. This helps alleviate concerns related to potential confounding and the reversal of causation (8). In contemporary times, numerous MR studies on intracranial aneurysms have emerged, contributing valuable clinical evidence (9, 10). This highlights that MR serves as a reliable research method capable of addressing diverse inquiries.

To clarify the potential influence of sexual variables on intracranial aneurysms, a two-sample MR study was conducted bidirectionally. One MR study delved into the association between sexual behaviors and major depressive disorder (MDD), investigating the potential impact of sexual variables on mental health (11). Hence, our objective was to scrutinize the relationship between the age at first sexual encounters and the number of sexual partners concerning intracranial aneurysms.

## 2 Supplies and procedures

### 2.1 Research design

**Figure 1:**
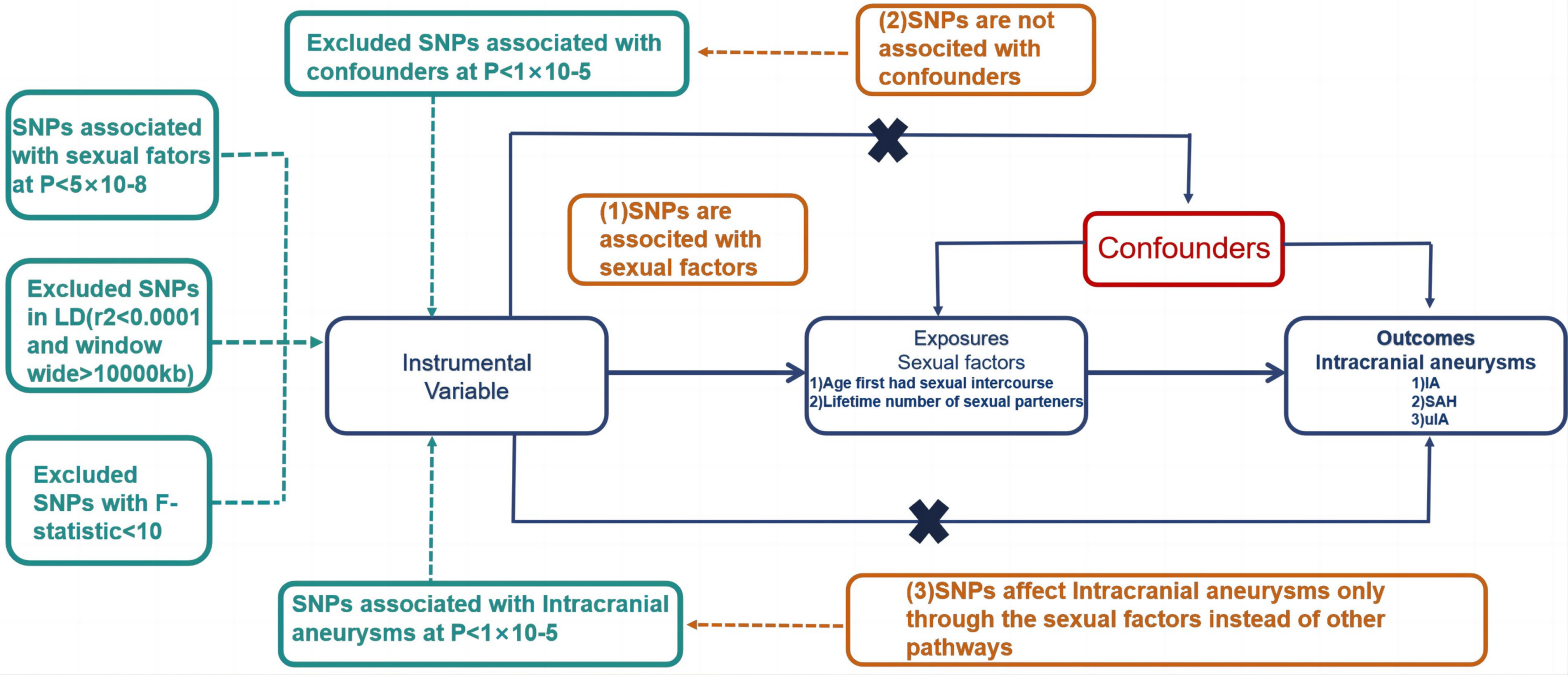
Visual representation of the study structure in MR. Illustration of the core principles guiding the study. MR; SNP, Single Nucleotide Polymorphism.

To scrutinize the associations involving sexual variables and intracranial aneurysms, we implemented a two-sample MR design in the current investigation. In order to assess the precise impact of two sexual variables—namely, the age at first sexual intercourse and the lifetime number of sexual partners—on intracranial aneurysms, we conducted a bidirectional two-sample MR study. This encompassed three sets of exposure data. To alleviate bias arising from the convergence of data, we compiled consolidated genetic data pertaining to sexual factors and intracranial aneurysms from publicly available databases. The study design (12) is explicated in the study framework diagram presented in Figure 1.

The three pivotal instrumental variable assumptions are detailed as follows:

1. Association with Exposure (Sexual Factors): Gene variants must exhibit a clear association with exposure circumstances (sexual variables). SNP selection follows strict criteria, including an upper limit of r2 not exceeding 0.001 within a 10,000 kb zone to eliminate linkage disequilibrium (LD) and significance at the genome-wide level (*p* < 5 × 10^−8^).
2. Independence from Other Factors: Genetic variants should not display associations with any other factors linked to both sexual variables and the relationship between Intracranial Aneurysms.
3. Exclusion from Unrelated Conditions: Genetic variants should not be correlated with intracranial aneurysms, except through sexual variables, with a genome-wide significant prerequisite of *p* < 5 × 10^−5^.

Trajectories diverging from these assumptions are illustrated with dashed lines.

### 2.2 Data sources

The recently concluded genome-wide association study (GWAS) conducted by the UK Biobank provided predictive biological indicators for two sexual variables within its extensive dataset. The study included the age at first sexual intercourse (406,457 individuals) and the lifetime number of sexual partners (378,882 individuals), with these variables reflecting the population sizes under investigation. Additionally, a meta-analysis of GWAS focused on intracranial aneurysms produced predictive biological markers specifically related to this medical condition. This comprehensive analysis involved 5,140 cases, encompassing both unruptured intracranial aneurysms and aneurysmal subarachnoid hemorrhages (aSAH), alongside 71,934 controls. The dataset was compiled from 23 cohorts, featuring individuals of European ancestry.

### 2.3 Standard protocol approval and patient consent

All data utilized in this MR study originated from GWAS summary data. It is crucial to note that ethical considerations and subject commitment were deemed unnecessary for the current analysis, as these aspects had already been appropriately addressed and secured for each of the original GWAS studies.

### 2.4 Statistical analysis

To assess the causality of intracranial aneurysm and sexual factors, we employed three distinct methods: MR-Egger, Inverse Variance Weighted (IVW), and Weighted Median. The IVW approach, widely recognized for its analysis without an intercept component, utilizes the inverse value of the result’s average margin of error as a weighting factor. This approach provides a comprehensive evaluation of the impact of the age at first intimate interactions and the number of sexual partners over a lifetime on the occurrence of IA. Ensuring the absence of pleiotropy in the selected Single Nucleotide Polymorphisms (SNPs) is crucial when employing the IVW method; otherwise, the outcomes could be substantially skewed.

The MR-Egger technique is utilized to furnish robust causal estimations that withstand deviations from standard IV assumptions and possess the capability to identify potential violations of these assumptions (13). The weighted median approach consolidates information from diverse genetic variants to produce a unified estimate of causation, ensuring consistency even when half of the instrumental variables exhibit null effects (14). We applied the MR-Egger intercept analysis to determine whether biological pleiotropy existed (15). Furthermore, to examine the likely occurrence of horizontal pleiotropy among the selected SNPs, we conducted MR-Egger regression. Additionally, leave-one-out assessment was carried out to examine the influence of each particular SNP and evaluate the accuracy of the causality assumptions.

The analyses were conducted using TwoSampleMR (version 0.5.6) and R software (version 4.2.3), with a predefined significance threshold set at 0.05 for the two-sided *p*-value.

## 3 Results

### 3.1 Association of lifetime sexual partners on the likelihood of intracranial aneurysm

**Figure 2:**
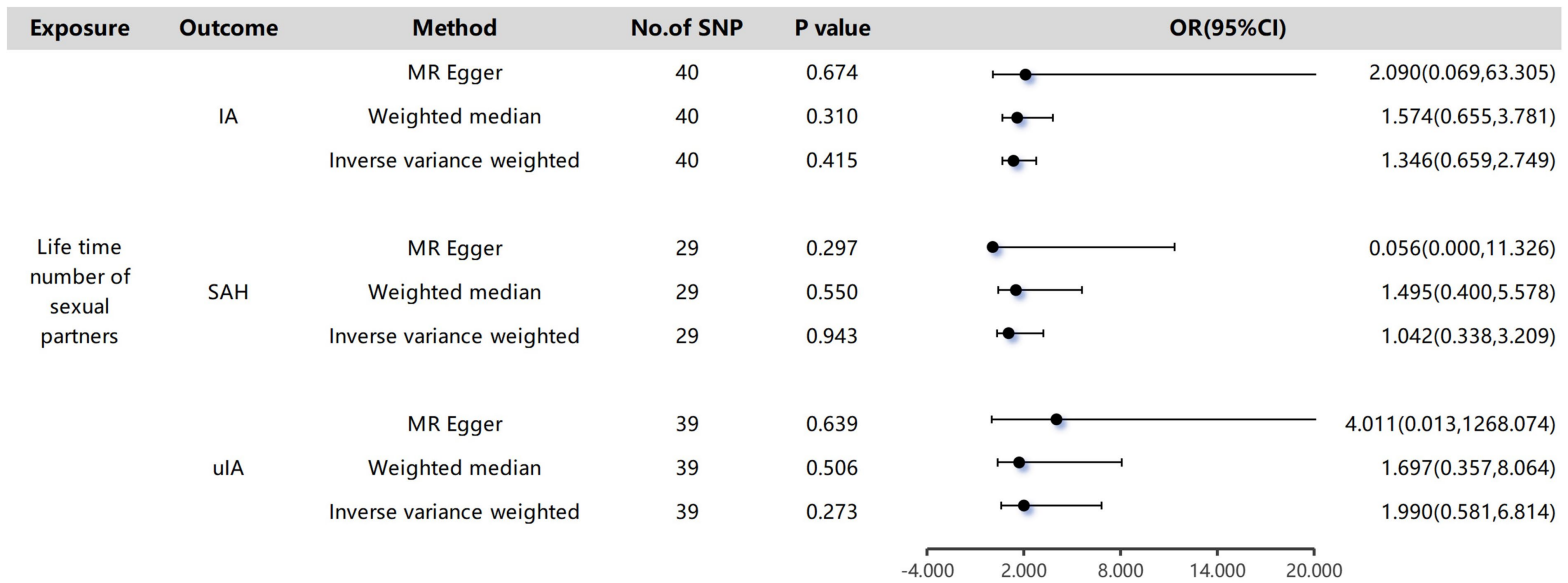
The causality of lifetime number of sexual partners and Intracranial aneurysms. The odds ratios are presented for each 1-standard deviation (SD) increase in genetically determined visceral adipose tissue (VAT) mass; CI, confidence interval.

In a two-sample MR analysis, there was limited indication of a potential association between the number of lifetime sexual partners and the occurrence of intracranial aneurysm. Utilizing the IVW method, the results showed odds ratios (OR) for the IA group (OR: 1.346, P: 0.415, 95% CI: 0.659–2.749), SAH group (OR: 1.042, P: 0.943, 95% CI: 0.338–3.209), and uIA group (OR: 1.990, P: 0.273, 95% CI: 0.581–6.814; Figure 2). The risk estimations were consistent, yielding comparable outcomes through MR-Egger regression and the Weighted Median methods.

### 3.2 Effect of age at first sexual intercourse on intracranial aneurysm

**Figure 3:**
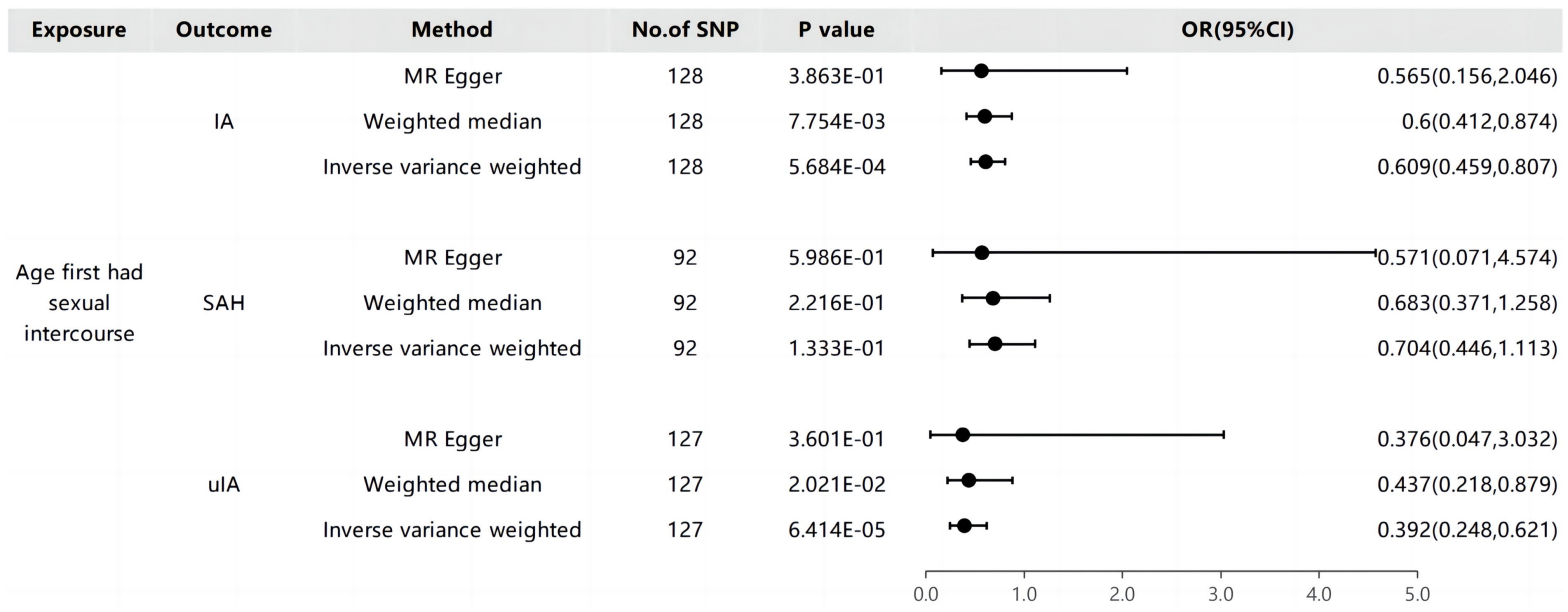
The causality of age first had sexual intercourse and Intracranial aneurysms.

**Figure 4:**
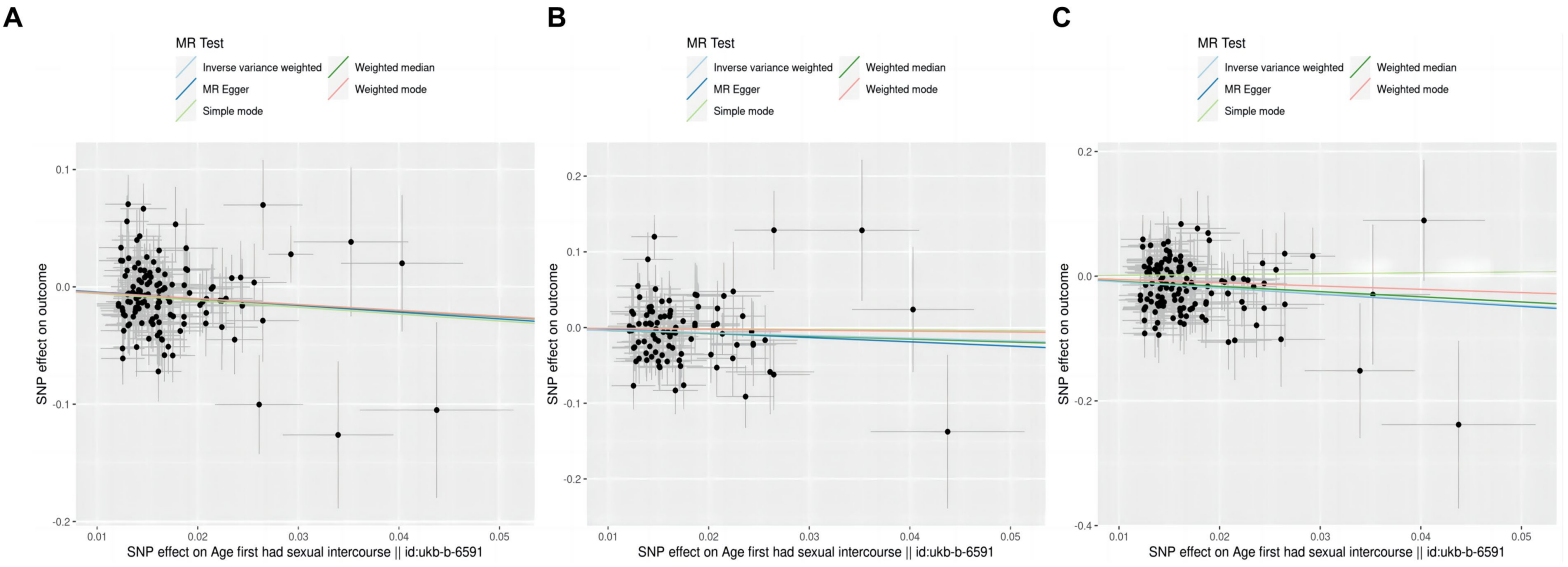
A diagram of the causative relationship between AFHS and intracranial aneurysm; **(A)**, AFHS and IA; **(B)**, AFHS and SAH; **(C)**, AFHS and uIA.

The outcomes derived from the IVW technique indicated a significantly reduced likelihood of IA and uIA in individuals who experienced early sexual intercourse (IA group: OR: 0.609, 95% CI: 0.459–0.807, P: 5.684E-04 with each genetically anticipated one-SD elevation). However, the MR-Egger and Weighted Median methods provided more cautious assessments that did not reach statistical significance (IA group: MR-Egger: OR: 0.565, P: 0.3863, 95% CI: 0.156–2.046; Weighted Median: OR: 0.6, P: 0.007754, 95% CI: 0.412–0.874; uIA group: MR-Egger: OR: 0.376, P: 0.3601, 95% CI: 0.047–3.032; Weighted Median: OR: 0.437, P: 0.02021, 95% CI: 0.218–0.879; Figure 3). The study findings suggest that the presumed effects of the age first had sexual encounters were consistently similar across the IVW and Weighted Median methods for overall intracranial aneurysms, despite some variations in statistical significance among the methods (Figure 4).

**Figure 5:**
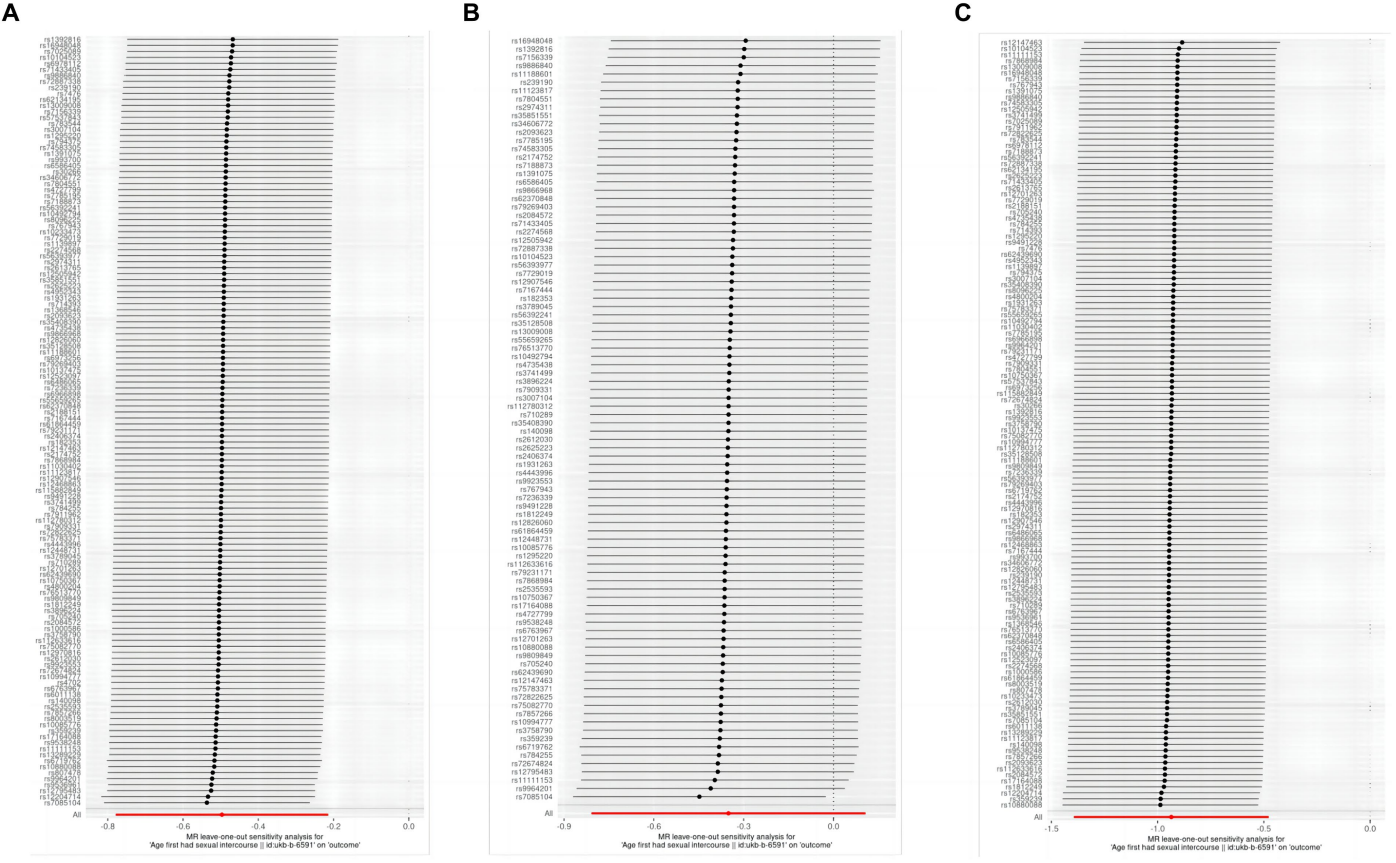
Evaluation of the figures for AFHS and intracranial aneurysm with a leave-one-out method; **(A)**, AFHS and IA; **(B)**, AFHS and SAH; **(C)**, AFHS and uIA.

The thorough evaluations performed in this study to assess the stability of identified causal effects consistently yielded significant results. All MR sensitivity analyses reached statistical significance, and the MR-Egger regression’s intercept test indicated minimal directed pleiotropy in the age first had sexual encounters group (IA group: *p*-value: 0.908; uIA group: *p*-value: 0.967). Moreover, the analysis of excluding one Single Nucleotide Polymorphism (SNP) at a time and individual SNP examination revealed that no specific site was identified to have a substantial impact on the outcomes (Figure 5).

## 4 Discussion

GWAS have successfully identified a multitude of genetic loci associated with various complex characteristics, conditions, and health conditions (16). The GWAS approach leverages the correlation structure of linkage disequilibrium (LD) within the genome, allowing cost-effective genotyping of a diverse range of variants. Non-genotyped variants can be imputed using a comprehensive genotyping reference dataset (17). However, the LD architecture also implies that identified associations often span across genomic regions containing multiple genes. Prioritizing among these genetic elements to identify the most functionally relevant genes based solely on the GWAS database poses a significant challenge. Given the vast number of putative causative variations within a typical genome-wide significant locus, laboratory-based investigation of relevant sections is both cost-prohibitive and impractical. The objective of this research is to investigate the relationship between sex-related characteristics, including age at first sexual intercourse and lifetime partners, and the probability of IA, aiming to understand the cause-and-effect dynamics involved. The findings consistently indicate that the age at first sexual intercourse has a favorable causal relationship with the occurrence of IA, with a notable impact on uIA. These outcomes hold strong across various MR methods. Importantly, this study marks the first-ever MR investigation delving into the correlation between sexual variables and IA.

Previous investigations have explored the potential association between sexual activities and aSAH, with researchers evaluating various physiological parameters such as blood pressure, heart rate, respiration rate and other parameters. Their findings indicated a growing acknowledgment of the potential role of each of these factors as triggers for the rupture of intracranial aneurysms (18). A seminal experiment conducted by Masters and Johnson (19) recorded physiological changes during coitus, contributing to advanced scientific and clinical understanding and providing nuanced insights into the physiological changes associated with the human sexual response. Over the past decades, engaging in sexual intercourse has been widely recognized as an independent contributor to the risk of both intracranial aneurysm and stroke (20, 21). However, our results suggest that premature sexual intercourse is instead a positive factor for IA. Earlier investigations often focused on the sexual activities of aSAH patients in the year preceding disease onset or explored the frequency of sexual intercourse. For example, Ebrahim et al. (22) analyzed the sexual frequency among middle-aged men and concluded that sexual intercourse is unlikely to be a contributing factor to strokes. In contrast, our study specifically focused on premature sexual intercourse and the number of sexual partners. Early sexual intercourse often occurs alongside alcohol consumption (6), and therefore, the correlation between the age of first sexual intercourse and aneurysm does not eliminate the potential influence of concurrent behaviors, such as smoking, acting as a confounding factor. Mark (23) employed a MR study to explore the influence of the age of initial intimate encounters and the quantity of sexual partners on oropharyngeal cancer (OPC) risk. The study uncovered a protective effect associated with a later age of first sexual intercourse while indicating an adverse influence connected to a higher number of sexual partners. However, complex correlations with other potential risk factors were identified. In multivariable analyses considering smoking and alcohol consumption, these effects were attenuated, emphasizing the need to account for confounding factors in understanding the correlation between sexual behaviors and OPC prevalence rate. A study conducted at Oxford identified 371 genetic markers associated with the age of initial intimate encounters and birth, providing insights into the fundamental causes of correlated traits, pleiotropy, and the interconnections between human reproductive behavior and the risk of disease (24). This suggests that collaborative efforts that integrate genetic, behavioral, and physiological data while maintaining the highest standards of privacy protection are essential to unraveling the multifaceted relationship between sexual behaviors and cerebrovascular health.

Returning to the formation of IA, it is a partially comprehended, gradual procession. Based on our current knowledge, deleterious hemodynamic elements such as increased hemodynamic pressure and factors that elevate the risk of vascular diseases—such as hypertension, lipid buildup, arteriosclerosis, and tobacco consumption—combined with genetic susceptibility, collectively contribute to the development of aneurysms (25–28). IA does not arise from congenital disorders but rather evolves over one’s lifetime, being particularly rare in individuals under 20 years of age. The probability of an uIA is higher in patients with FDRs classified as ADPKD, an intracranial aneurysm, or a SAH (29). Robe’s MR study revealed a noteworthy connection between genetic predisposition to elevated levels of sex hormone-binding globulin (SHBG) and an elevated risk of aSAH among women. Conversely, the study also discovered that the risk of aSAH was not significantly affected by genetically determined increased age at menarche (AAM) or age at menopause (30). However, it is important to clarify that this study does not advocate for early sexual activity as a preventive measure for IA. Historical contexts where early fertility was common suggest a biological adaptability to early sexual maturity, which may carry different implications in contemporary settings. Our study aims to contribute to the understanding of potential biological mechanisms and not to suggest changes in social or medical guidelines without further comprehensive studies. In a recent study, researchers explored the relationship between genetically predicted elevated levels of SHBG and kidney dysfunction. Interestingly, they found that higher genetically predicted SHBG was linked to a decreased likelihood of chronic kidney dysfunction (CKD) and improved renal function, and this association was evident only in men, not in women. This suggests a potential gender-specific role of SHBG in CKD, emphasizing its impact in men (31). These findings imply a nuanced relationship between SHBG and health outcomes, with SHBG showing a protective association in men concerning CKD and, conversely, an increased risk of aSAH among women. A similar protective effect can be observed in the connection between genetically predicted SHBG and ischemic heart disease (IHD) in men. The absence of an association in women may be attributed to a smaller effect size, potentially beyond the detection capability of the current investigations. A larger sample of women would be beneficial for replication (32). Importantly, these observations align with broader discussions in the literature (29, 33, 34), where some genetic kidney diseases are often found in conjunction with IA. This interplay of genetic factors, hormonal regulation, and kidney diseases further highlights the intricate connections within the vascular system, emphasizing the need for a comprehensive understanding of genetic influences on various health conditions. Secondly, given that genetic variants account for only a small fraction of the variability in SHBG, MR research demands substantial sample sizes (35), a criterion fulfilled through the utilization of the UK Biobank.

Despite the intriguing findings in our MR study, it is essential to acknowledge the inherent uncertainties surrounding the causal link between sexual intercourse and IA. Our investigation primarily focused on premature sexual intercourse, and the lack of detailed information on age and exact numbers of sexual partners poses notable limitations. Preserving the privacy of sensitive information emerged as a key challenge in our study. It is important to note that our dataset from GWAS does not include direct measurements of sexual activity frequency, which is a limitation of this study. As a result, we were unable to delve into the granularity of age-related variations or ascertain the specific impact of varying numbers of sexual partners on IA risk. This limitation underscores the delicate balance researchers must strike between extracting meaningful insights and preserving the confidentiality of sensitive data. Future studies with more extensive datasets and perhaps innovative privacy-preserving methodologies could provide a more nuanced understanding of these associations.

## 5 Conclusion

In summary, this MR study represents the inaugural attempt, to our knowledge, to investigate potential links between sexual factors and IA. Premature sexual intercourse appears to decrease the risk of uIA. Despite the utility of MR in reducing confounding, our study is not exempt from limitations. MR assesses lifetime effects, unlike short-term interventions, potentially impacting the comparability of effects on IA. Additionally, the predictive power of genetic variants for premature initial sexual encounters is modest, necessitating larger sample sizes (35), which we achieved using the UK Biobank. These limitations emphasize the need for careful interpretation and suggest avenues for improvement in future research.

## Data availability statement

The original contributions presented in the study are included in the article/Supplementary material, further inquiries can be directed to the corresponding author.

## Ethics statement

Ethical review and approval was not required for the study on human participants in accordance with the local legislation and institutional requirements. Written informed consent from the patients/participants or patients/participants' legal guardian/next of kin was not required to participate in this study in accordance with the national legislation and the institutional requirements.

## Author contributions

PW: Writing – original draft, Conceptualization, Investigation, Methodology, Writing – review & editing, Supervision. PA: Writing – original draft, Methodology, Software, Visualization. KK: Writing – review & editing. MA: Writing – review & editing. XC: Writing – review & editing. ZW: Writing – review & editing. AM: Software, Visualization, Writing – review & editing.
