# Supplementary material for: Early sexual activity lowers the incidence of intracranial aneurysm: a Mendelian randomization investigation
Source: Front Neurol. 2024 Jun 4;15:1349137. doi: 10.3389/fneur.2024.1349137 (PMC11184162; doi:10.3389/fneur.2024.1349137)
Supplement: Supplementary file 4 [file Data_Sheet_4.PDF]

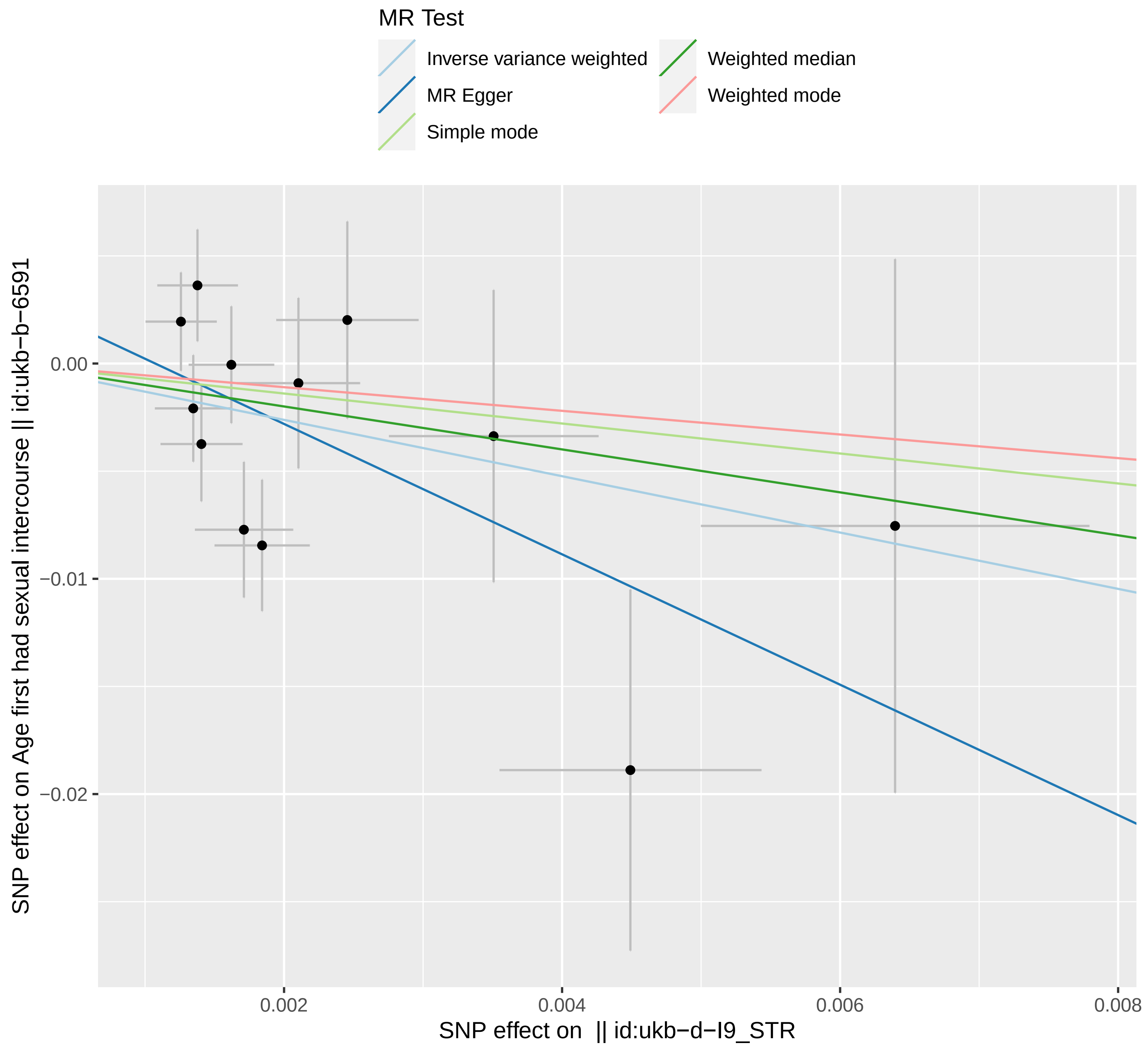
MR Test
Inverse variance weighted
MR Egger
Simple mode
Weighted median
Weighted mode
0.00
−0.01
−0.02
0.002
0.004
0.006
0.008
SNP effect on Age first had sexual intercourse || id:ukb−b−6591
SNP effect on || id:ukb−d−I9_STR
