# Supplementary figures and images for: Early sexual activity lowers the incidence of intracranial aneurysm: a Mendelian randomization investigation
Source: Front Neurol. 2024 Jun 4;15:1349137. doi: 10.3389/fneur.2024.1349137 (PMC11184162; doi:10.3389/fneur.2024.1349137)

# MR Method

- Inverse variance weighted
- MR Egger

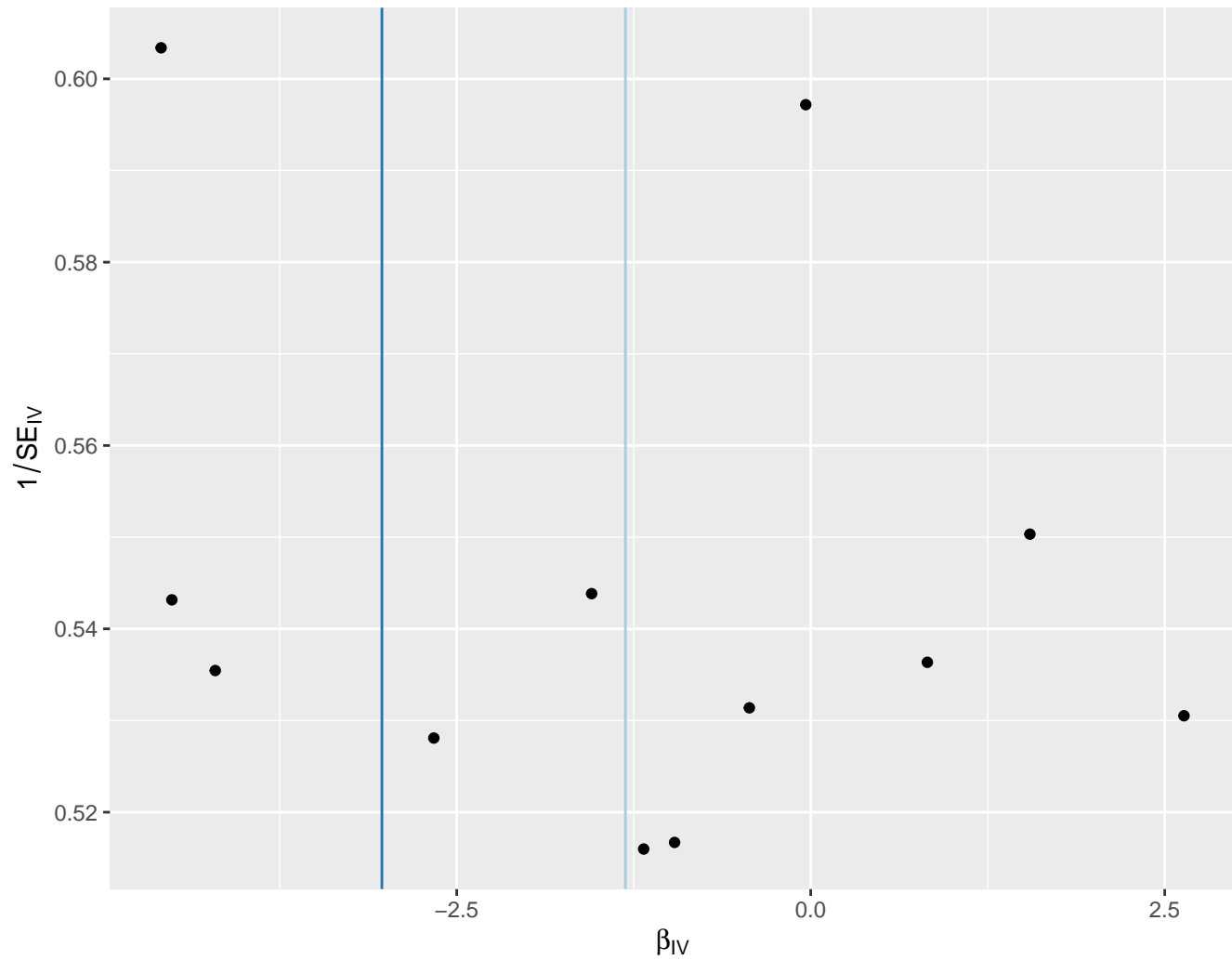

Supplement: Supplementary file 5 [file Data_Sheet_5.PDF]

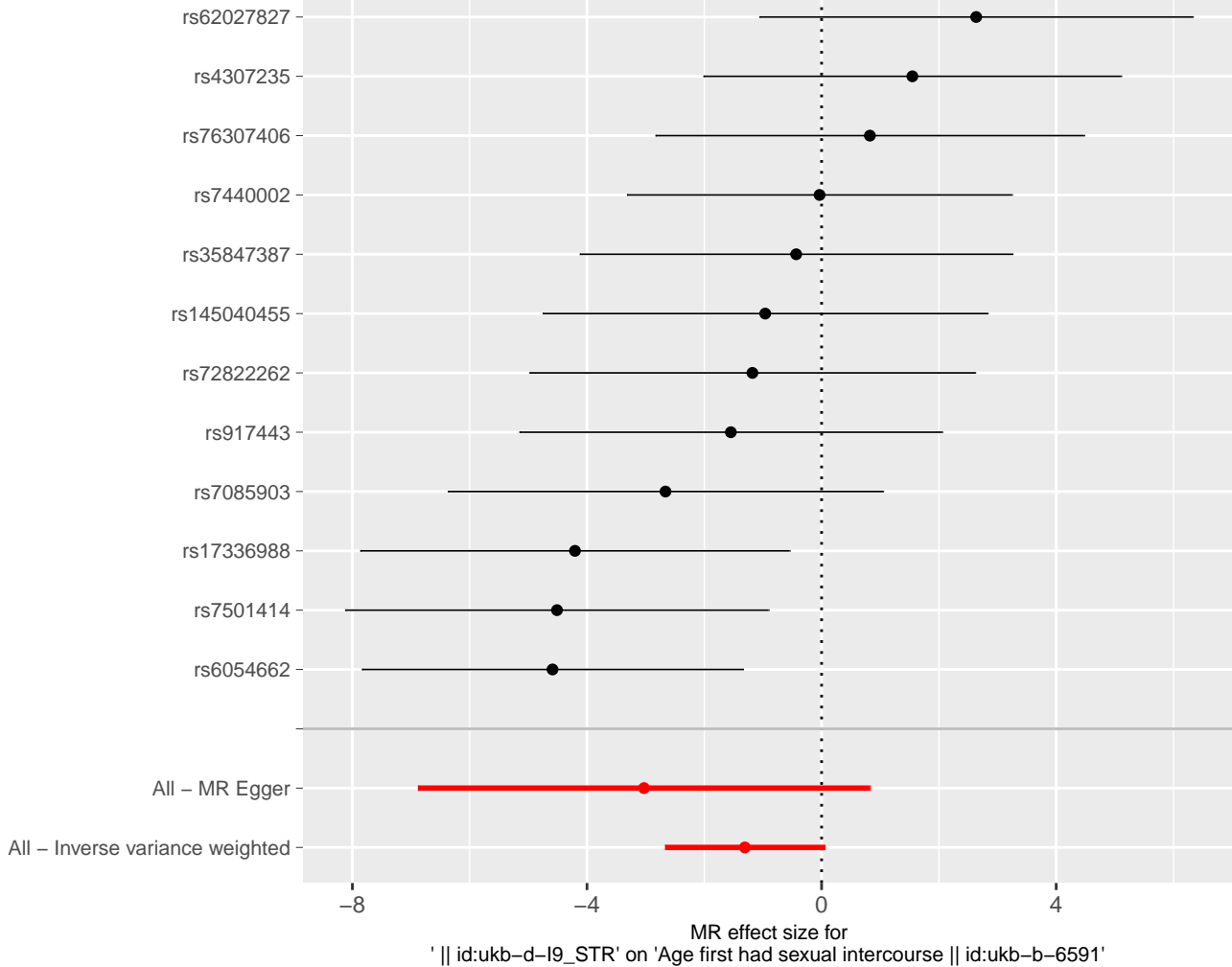

Supplement: Supplementary file 6 [file Data_Sheet_6.PDF]
