# Supplementary material for: Early sexual activity lowers the incidence of intracranial aneurysm: a Mendelian randomization investigation
Source: Front Neurol. 2024 Jun 4;15:1349137. doi: 10.3389/fneur.2024.1349137 (PMC11184162; doi:10.3389/fneur.2024.1349137)
Supplement: Supplementary file 7 [file Data_Sheet_7.PDF]

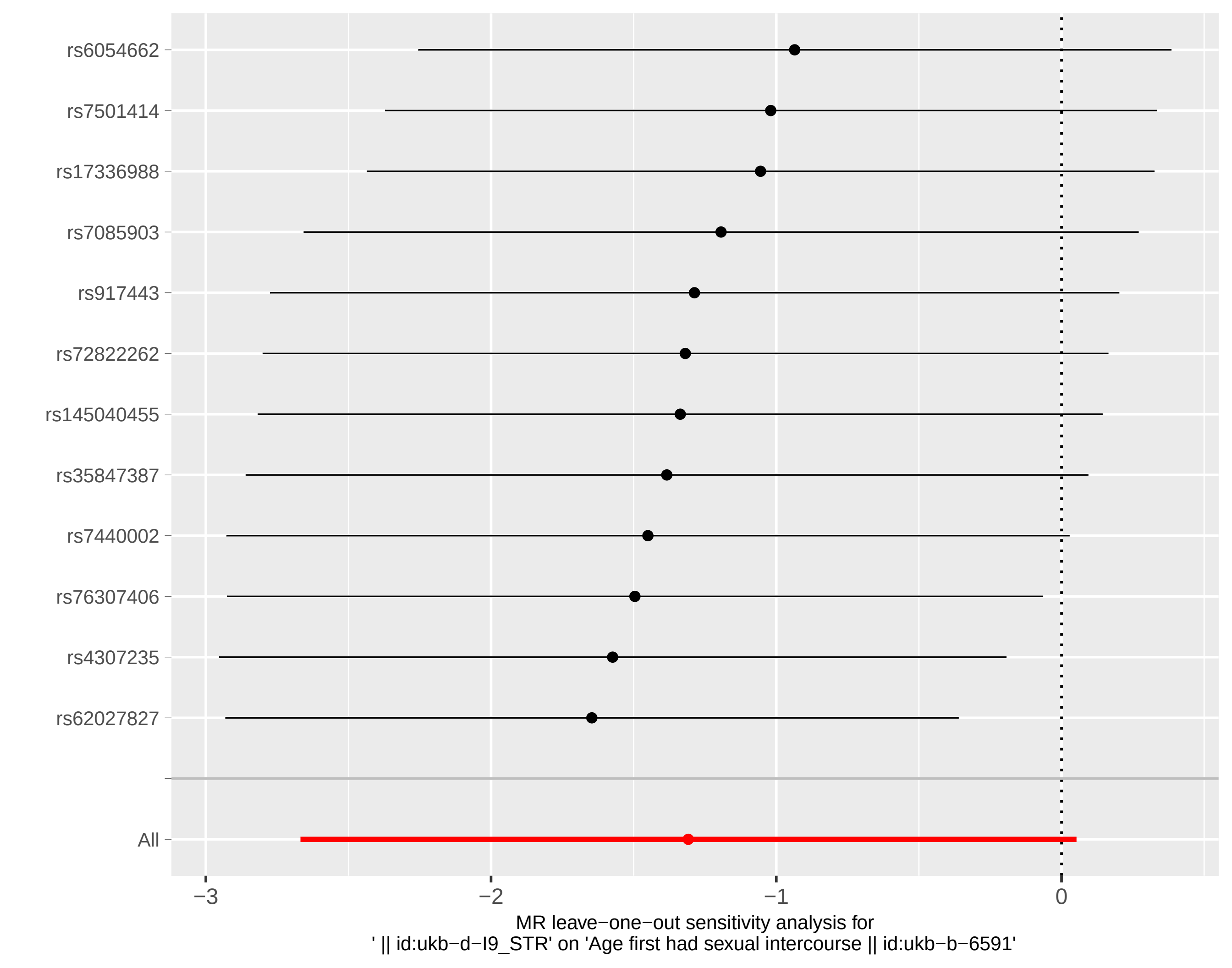

rs6054662
rs7501414
rs17336988
rs7085903
rs917443
rs72822262
rs145040455
rs35847387
rs7440002
rs76307406
rs4307235
rs62027827
All
−3
−2
−1
0
MR leave−one−out sensitivity analysis for
' || id:ukb−d−I9_STR' on 'Age first had sexual intercourse || id:ukb−b−6591'
